# Psychometric properties of the eating disorder examination-questionnaire in Japanese adolescents

**DOI:** 10.1186/s13030-017-0094-8

**Published:** 2017-04-04

**Authors:** Tomoyo Mitsui, Toshiyuki Yoshida, Gen Komaki

**Affiliations:** 1 Department of Psychology, Faculty of Human Development and Education, Kobe Shinwa Women’s University, 7-13-1 Suzurandai-Kitamachi, Kita-ku, Kobe, Hyogo Japan; 2 School of Health Sciences, Fukuoka, International University of Health &Welfare, 137-1 Enokizu, Ohkawa, Fukuoka Japan

**Keywords:** EDE-Q, Psychometric Properties, Fear of Obesity, Self-esteem, Factor structure, Convergent validity, Adolescents

## Abstract

**Background:**

Although the Eating Disorder Examination Questionnaire version 6.0 (EDE-Q) is one of the most widely used questionnaires for eating disorders in Western countries, no research has addressed the psychometric properties of the EDE-Q in a Japanese sample.

**Methods:**

We explored the factor structure of the EDE-Q and examined the internal consistency of the derived scales for Japanese participants (Study I), the convergent validity with other eating disorder-related psychological measures (Study II) and the distinction between the derived two body image-related factors with psychological measures (StudyIII). The EDE-Q was administered to 1,430 undergraduate students in Study I and in Study II was subsequently assessed by two self-report measures of eating pathology, the Eating Attitudes Test (EAT-26) for 558 undergraduate students and the Eating Disorders Inventory-II (EDI-II) 111. In StudyIII, another 225 undergraduate students participated in an examination of the relationships of the derived body image-related subscales of the EDE-Q with the psychological measures of the Rosenberg Self-Esteem Scale, Beck Depression Inventory, Public Self-Consciousness Scale, and Multidimensional Perfectionism Scale.

**Results:**

Exploratory factor analysis of the EDE-Q identified four meaningful factors. Of the original four EDE-Q factors, “Restriction” and “Eating Concern” were retained. However, the other two factors, “Shape” and “Weight” Concerns, were combined into two different factors: “Fear of Obesity” and “Self-Esteem Based on Shape and Weight”. Internal consistency of the derived four factors was adequate, and the relationships with EDI-II and EAT-26 measures demonstrated convergent validity. Analysis of the distinction between “Fear of Obesity” and “Self-Esteem Based on Shape and Weight” revealed that only “Self-Esteem Based on Shape and Weight” was significantly associated with the measures assessing psychopathology related to eating disorders.

**Conclusions:**

This study describes restructured factors of the EDE-Q that were tested with undergraduate students. The distinction between two factors, “Fear of Obesity” and “Self-Esteem Based on Shape and Weight”, may further the understanding of the psychopathology of the eating disorders of adolescent Japanese subjects to facilitate future developments in research and treatment.

## Background

An alarming increase in the number of teenagers with eating disorders has recently been reported in Japan [1]. Although several translated self-report measures of eating pathology, such as the Eating Disorders Inventory-II (EDI-II) [2] and the Eating Attitude Test-26 (EAT-26) [3] are available, they have certain limitations [4–7] for Japanese subjects, such as a high rate of false negative results for the detection of Bulimia Nervosa for EAT-26 [6] and the large number of items (91i) of EDI-II [7].

The Eating Disorder Examination-Questionnaire (EDE-Q) [8] is a self-report measure of eating disorder symptoms that was derived from the Eating Disorder Examination interview (EDE) [9]. Although the EDE-Q is one of the most widely used questionnaires for eating disorders in Western countries [10], no research has addressed the psychometric properties of the EDE-Q in a Japanese sample.

The original version of the EDE-Q contains attitudinal questions in the form of four subscales: Restraint, Eating Concern, Shape Concern, and Weight Concern [8]. The remaining questions assess the frequency of episodes of objective binge eating, self-induced vomiting, laxative misuse, diuretic misuse, and driven exercise for weight control. Research has found that the EDE-Q has good internal consistency [11–13] and temporal stability [11, 12, 14], as well as high convergent validity [15–19], discriminant validity, and sensitivity to change [18, 20, 21].

Despite these generally favorable psychometric properties [11–21], recent studies of the EDE-Q have not provided support for the theorized four-factor structure [13, 22–29]. Several studies have suggested that the shape and weight concern items may in fact contribute to a single factor [13, 23, 26–29]. Support for a three-factor model (restraint, eating-related concerns, and shape and weight concerns) has been found in clinical samples using exploratory factor analysis (EFA) [23, 26, 29].

In addition, it remains unknown whether preoccupation with thinness and shape and weight concerns play a central role in the etiology of eating disorders in Japan, as compared with the situation in Western countries [30]. For example, recent studies using the EAT-26 [3] and the EDI-II [2] to investigate attitudes and psychopathology of eating disorders have produced results that are somewhat different between Asian and Caucasian adolescent girls [31, 32]. Furthermore, studies comparing Japanese adolescents with North Americans and Europeans in terms of drive for thinness and body dissatisfaction have reported mixed findings; one group [33] found that female Japanese without eating disorders reported higher levels of drive for thinness and body dissatisfaction than their German counterparts, whereas another group [30] found lower levels of drive for thinness and body dissatisfaction among Japanese than among a North American-referenced comparison. Thus, EDE-Q scores among Japanese adolescents should be examined with respect to the cultural context that affects attitudes such as shape and weight concerns while taking gender into account.

Hrabosky et al. [22], Peterson et al. [13], and Grilo et al. [34] emphasized the importance of the distinction between “body image dissatisfaction” and “overvaluation of shape and weight” with regard to the factor structure of the EDE-Q. Hrabosky et al. suggested that “overvaluation of shape and weight” appears to be a construct related to, but distinct from, “body image dissatisfaction.” In these studies, “overvaluation of shape and weight” is considered more pathological than “body image dissatisfaction,” and it therefore differentiates patients with eating disorders from normal controls [22]. For this reason, psychological measures that are strongly associated with the etiology of eating disorders are useful to distinguish those body image related factors and at the same time to explore the factor structure of the EDE-Q.

In this context, we used EFA to investigate the factor structure of the EDE-Q in a group of Japanese undergraduate students and examined the internal consistency of the derived factors (Study I), the convergent validity with other eating disorder-related psychological measures (Study II), and the distinction between the two derived body image-related factors and psychological measures (StudyIII).

## Study I

In Study I, we explored the factor structure of the EDE-Q in Japanese undergraduate students by means of EFA, as well as the internal consistency of the derived scales.

### Methods

#### Participants

Participants were undergraduate students (*N* = 1,430, 1,169 female and 261 male) recruited from seven different universities located in suburban areas of Japan. They completed a 28-item Japanese translation of the Eating Disorder Examination-Questionnaire (EDE-Q) 6.0. A total of 1,632 questionnaires were distributed between October 2011 and November 2012. Seventy-eight participants declined to participate and 124 returned uncompleted questionnaires. The response rate was thus 95.2%; the valid response rate was 92.0%.

The mean age of the sample was 19.4 years (*SD* = 1.3), and the mean BMI was 20.6 kg/m^2^ (*SD* = 2.7).

### Measures

#### Eating Disorder Examination–Questionnaire; EDE-Q

The translation of the EDE-Q 6.0 [35] into Japanese was carefully done to insure that there were no differences in nuance between the original English and the new Japanese version. The version translated to Japanese was back-translated into English by a person who is fluent in both English and Japanese. The back-translated version of the EDE-Q was then confirmed in terms of accuracy by the authors of the original English version.

The 28 item EDE-Q contains 22 attitudinal questions that assess the severity of eating pathology over the previous 28 days, with participants responding on a seven-point rating scale ranging from 0 = *none* to 6 = *every day*, with higher scores reflecting either greater severity or frequency. In the original version, these attitudinal questions comprise four subscales: Restraint, Eating Concern, Shape Concern, and Weight Concern.

### Statistical analyses

An exploratory factor analysis (EFA) of the 22 attitudinal items of the EDE-Q was conducted to check the consistency of its factorial structure with that of the original version. To choose the number of factors for extraction, an “eigenvalue >1” criterion [36] and scree plot identification [37] were used. The percentage of missing responses was very low at 4.8%, and only 8% of the participants had missing values for one or more of the 22 items. A significance level of *p* < 0.05 was adopted. Statistical analyses were carried out using SPSS 20.0 in all studies.

### Ethical considerations

All studies were approved by the institutional ethics committee of the International University of Health &Welfare (No. 13-Io-180), and all participants provided written informed consent prior to completing the questionnaire package. All surveys were conducted during regular class sessions.

## Results

Eigenvalues decreased from 8.60 to 2.39, 1.64, 1.10, and 1.04. We first examined whether we could obtain the same four factor structure as that found in the original EDE-Q [8]. EFA identified four factors with the principal component method and equimax rotation. The four factors accounted for 62.17% of the total variance. All items loading above 0.35 were retained. These items and their loadings on each factor are displayed in Table 1.

**Table 1:** Pattern matrix of the principal axis factor analysis of EDE-Q attitudinal items (*n* = 1430)

	Factor 1 Fear of Obesity	Factor 2 Self-Esteem Based on Shape and Weight	Factor 3 Restriction	Factor 4 Eating Concern
EDE-Q item				
Desire to lose weight (WC)	**0.78**	0.25	0.31	0.11
Feeling of fatness (SC)	**0.75**	0.30	0.23	0.09
Dissatisfaction with weight (WC)	**0.73**	0.53	0.15	0.04
Dissatisfaction with shape (SC)	**0.70**	0.56	0.11	0.01
Flat stomach (SC)	**0.69**	0.04	0.21	0.23
Discomfort about seeing body (SC)	**0.62**	0.57	0.03	0.11
Fear of weight gain (SC)	**0.55**	0.19	0.28	0.43
Importance of shape (SC)	0.23	**0.79**	0.25	0.12
Importance of weight (WC)	0.26	**0.78**	0.27	0.14
Discomfort about exposure (SC)	0.56	**0.62**	0.06	0.07
Reaction to weighting (WC)	0.26	**0.57**	0.02	0.23
Social eating (EC)	−0.03	**0.55**	0.05	0.47
Dietary rules (R)	0.09	0.10	**0.82**	0.08
Food avoidance (R)	0.12	0.13	**0.81**	0.12
Restraint over eating (R)	0.22	0.15	**0.79**	0.06
Avoidance of eating (R)	0.07	0.00	**0.53**	0.34
Empty stomach (R)	0.33	0.05	**0.42**	0.41
Preoccupation with weight and shape (WC/SC)	0.06	0.09	0.17	**0.78**
Preoccupation with food, eating or calories (EC)	0.03	0.05	0.22	**0.77**
Fear of losing control over eating (EC)	0.22	0.12	0.11	**0.71**
Guilt about eating (EC)	0.38	0.37	0.21	**0.41**
Eating in secret (EC)	0.02	0.23	0.02	**0.37**
Eigenvalue	8.60	2.39	1.64	1.10
Percentage of Variance	19.17	16.43	13.50	13.07
Cronbach’s alpha	0.91	0.82	0.78	0.71

Original subscale allocations are shown in brackets

*SC*: Shape Concern, *WC* Weight Concern, *EC* Eating Concern, *R* Restriction

Factor 1 included five items from the original Shape Concern subscale (e.g., feeling of fatness) and two items from the original Weight Concern subscale (e.g., desire to lose weight). Because these items focused on body dissatisfaction and the importance of shape and weight, Factor 1 was designated “Fear of Obesity,” and explained 19.17% of the total variance. Factor 2 contained two items from the original Shape Concern subscale (importance of shape, discomfort about exposure), two items from the original Weight Concern subscale (importance of weight, reaction to weighing) and one item from the original Eating Concern subscale (social eating). Because these items focused on the value of one’s shape and weight on self-worth, Factor 2 was designated “Self-Esteem Based on Shape and Weight,” and explained 16.43% of the total variance. Factor 3, containing all five items from the original Restraint subscale (e.g., dietary rules) retained the original name “Restraint”, and explained 13.5% of the total variance. Factor 4, containing the four items from the original Eating Concern subscale (e.g., preoccupation with food) and one item from both the Shape Concern and Weight Concern subscales related to preoccupation (e.g., preoccupation with weight and shape) was designated “Eating Concern,” and explained 13.07% of the total variance.

Cronbach’s coefficient alphas were computed for the individual subscales (n = 1,430) as follows: Fear of Obesity 0.91; Self- Esteem Based on Shape and Weight 0.82; Restraint 0.78; Eating Concern 0.71 (Table 1). These data show acceptable to good homogeneity of the subscale items.

Descriptive statistics, correlations, and gender differences among scale scores for the restructured EDE-Q and original EDE-Q are shown in Table 2. All four subscales were highly inter-correlated on both scorings. The correlation between “Fear of Obesity” and “Self-Esteem Based on Shape and Weight” was 0.75 (95% CI [0.73, 0.77]) in the restructured EDE-Q, whereas the correlation of Weight Concern and Shape Concern subscales was 0.91 (95% CI [0.90, 0.92]) in the original EDE-Q.

**Table 2:** Mean scores, standard deviations and correlations for both restructured and original EDE-Q subscales and global scores

	Correlations				Mean		
	2	3	4	Global score	Mean (SD)	Males Mean (SD)	Females Mean (SD)
restructured EDE-Q							
1. Fear of Obesity	0.75^***^	0.5^***^	0.52^***^	0.94^**^	2.75 (1.92)	1.10 (1.45)	3.12 (1.81)^**^
2. Self-Esteem Based on Shape and Weight		0.41^***^	0.52^***^	0.84^**^	1.67 (1.37)	0.75 (1.12)	1.87 (1.34)^**^
3. Restriction			0.48^***^	0.69^**^	0.88 (1.17)	0.41 (0.87)	0.99 (1.20)^**^
4. Eating Concern				0.71^**^	0.50 (0.80)	0.24 (0.59)	0.55 (0.82)^**^
Global score					1.57 (1.14)	0.67 (0.88)	1.10 (1.57) ^**^
original EDE-Q							
1. Weight Concern	0.91^**^	0.51^**^	0.59^**^	0.94^**^	1.95 (1.45)	0.77 (1.15)	2.20 (1.38) ^**^
2. Shape Concern		0.51^**^	0.6^**^	0.96^**^	2.25 (1.56)	0.96 (1.20)	2.54 (1.48)^**^
3. Restriction			0.47^**^	0.69^**^	0.88 (1.17)	0.41 (0.87)	0.99 (1.20)^**^
4. Eating Concern				0.71^**^	0.54 (0.80)	0.27 (0.64)	0.61 (0.82)^**^
Global score					1.52 (1.11)	0.65 (0.86)	1.71 (1.07)^**^

**p < 0.01, ***p < 0.001

Female participants showed significantly higher scores than males on “Fear of Obesity” (*U* = 15.91, *p* < 0.01), “Self-Esteem Based on Shape and Weight” (U =13.73, *p* < 0.01), “Restraint” (*U* = 10.72, *p* < 0.01), “Eating Concern” (*U* =8.93, *p* < 0.01), and global scores (*U* = 15.48, *p* < 0.01).

The original EDE-Q subscales showed similarly significant differences (“Weight Concern”: *U* = 15.53, *p* < 0.01; “Shape Concern”: *U* = 15.60, *p* < 0.01: “Restraint”: *U* = 10.72, *p* < 0.01: “Eating Concern”: *U* = 9.38, *p* < 0.01) and global scores (*U* = 15.42, *p* < 0.01) (Table 2).

## Study II

We examined the convergent validity of the factors of the EDE-Q derived from Study I with two other eating questionnaire measures, EAT-26 [3] and EDI- II [2].

### Methods

#### Participants

Participants were undergraduate students (*N* = 558; 469 female, 82 male, 7 sex unknown) to examine the correlations of the factors of the restructured EDE-Q with EAT-26. Mean age was 20.11 years (*SD* = 2.52) and mean BMI was 20.73 kg/m^2^ (*SD* = 2.77). The response rate was 96.5%, and the valid response rate was 95.9%. To examine the correlations with EDI- II, we recruited 111 female undergraduate students with a mean age of 18.52 years (*SD* = 0.77) and a mean BMI of 21.04 kg/m^2^ (*SD* = 3.04). The response rate was 100%, and the valid response rate was 90.1%.

### Measures

#### Eating Disorder Examination–Questionnaire; EDE-Q

Refer to Study I.

#### Eating Attitudes Test (EAT-26) [3]

The EAT-26 is a 26-item self-report questionnaire used to assess eating attitudes and behaviors. Each item is scored on a six-point scale ranging from 1 (*never*) to 6 (*always*). A higher score indicates greater levels of attitudes and behaviors related to ED. We calculated three subscales, Dieting, Bulimia and food preoccupation, and Oral control, as indicated in the EAT-26. The reliability and validity of the Japanese version of the EAT-26 have been demonstrated [4–6].

#### Eating Disorders Inventory-II (EDI-II) [2]

The EDI-II is a 91-item self-report questionnaire used to assess psychological and behavioral characteristics related to eating disorders. Each item is scored on a six-point scale ranging from 0 (*never*) to 5 (*always*). A higher score indicates greater levels of ED pathology. The three least pathological responses receive 0 points and the other responses 1, 2, and 3 to denote increasing severity. The reliability and validity of the Japanese version of the EDI-II have been demonstrated [7]. For this study, we used the subscales Drive for Thinness (concern with weight and dieting), Body Dissatisfaction, and Bulimia (binge and purge).

### Statistical analyses

Pearson’s correlation coefficients were used to evaluate the relationships between the subscale scores of the EDE-Q and those of the EAT-26 and EDI-II.

## Results

The convergent validity of the original and restructured EDE-Q was evaluated by examining whether or not the subscales scores correlated with measures of similar constructs, the EAT-26 and the EDI-II, respectively. Correlations between the original and restructured EDE-Q subscales and the EAT-26 and EDI-II subscales are shown in Tables 3 and 4.

**Table 3:** Correlations between EDE-Q restructured and original subscales and EAT-26 subscales

	EAT-26		
	Dieting	Bulimia and Food Preoccupation	Oral control
[EDE-Q restructured subscales]			
Fear of Obesity	0.64^**^	0.27^**^	−0.06
Self-Esteem Based on Shape and Weight	0.51^**^	0.28^**^	0.05
Restraint	0.61^**^	0.29^**^	0.12^**^
Eating Concern	0.58^**^	0.50^**^	0.14^**^
Global score	0.71^**^	0.37^**^	0.04
[EDE-Q original subscales]			
Weight Concern	0.63^**^	0.31^**^	0.01
Shape Concern	0.64^**^	0.31^**^	−0.02
Restraint	0.61^**^	0.29^**^	0.12^**^
Eating Concern	0.57 ^**^	0.50^**^	0.14^**^
Global score	0.71^**^	0.38^**^	0.04

**p < 0.01

**Table 4:** Correlations between EDE-Q restructured and original subscales and EDI-II subscales

	EDI-II		
	Drive for Thinness (DT)	Body Dissatisfaction (BD)	Bulimia (B)
[EDE-Q restructured subscales]			
Fear of Obesity	0.80^**^	0.69^**^	0.47^**^
Self-Esteem Based on Shape and Weight	0.69^**^	0.52^**^	0.50^**^
Restraint	0.46^**^	0.22^*^	0.22^*^
Eating Concern	0.41^**^	0.21^*^	0.52^**^
Global score	0.80^**^	0.59^**^	0.53^**^
[EDE-Q original subscales]			
Weight Concern	0.75^**^	0.59^**^	0.44^**^
Shape Concern	0.80^**^	0.66^**^	0.50^**^
Restraint	0.46^**^	0.22^*^	0.22^*^
Eating Concern	0.43^**^	0.23^*^	0.60^**^
Global score	0.79^**^	0.58^**^	0.52^**^

*p <0.05, **p < 0.01

Like the original subscales, the restructured EDE-Q subscales showed moderate correlations with the EAT-26 and EDI-II subscales. The subscales “Fear of Obesity” and “Self-Esteem Based on Shape and Weight” in the restructured EDE-Q were strong and moderately correlated with the two EDI-II subscales of body image, Drive for Thinness and Body Dissatisfaction.

## Study III

We further examined the relationships of the derived body image-related subscales of the EDE-Q, “Fear of Obesity” and “Self-Esteem Based on Shape and Weight”, with psychological measures that are generally related to the etiology of eating disorders [22, 38–40]: the Rosenberg Self-Esteem Scale, Beck Depression Inventory, Public Self-Consciousness Scale, and Multidimensional Perfectionism Scale.

### Methods

#### Participants

Participants were female undergraduate students (*N* = 225). Mean age was 19.6 years (*SD* = 1.0). Mean BMI was 20.9 kg/m^2^ (*SD =* 3.0). The response rate was 100% and the valid response rate was 97.8%.

### Measures

#### Eating Disorder Examination–Questionnaire; EDE-Q

Refer to Study I.

#### Rosenberg Self-Esteem Scale (RSES) [41]

The RSES is a 10-item self-report questionnaire used to assess self-esteem, comprising cognitive and affective aspects of self-worth. Each item is scored on a five-point scale ranging from 1 (*strongly disagree*) to 5 (*strongly agree*). A self-esteem score is calculated after reversing the positively worded items. A higher score indicates greater self-esteem. The reliability and validity of the Japanese translation of the RSES have been demonstrated [42].

#### Beck Depression Inventory Second Edition (BDI-II) [43]

The BDI-II is a 21-item self-report questionnaire used to assess severity of depression symptoms, based on the diagnostic criteria for depressive disorders in the DSM-IV. It is also useful for detecting psychosocial distress [44]. Each item is scored from 0 to 3, with higher score indicating greater intensity of the symptom. The total score is the sum of the items, ranging from 0 to 63; a higher total score indicates greater depression. The reliability and validity of the Japanese version of the BDI-II have been demonstrated [45].

#### Public Self-Consciousness Scale (PSS) [46]

Sugawara [47] constructed a Japanese version of the Self-Consciousness Scale [46] (26-item), which has two factors: Private Self-focus and Public Self-focus. In the present study, the Public Self-Consciousness Scale (11-item) was used to assess attention directed toward oneself from an external perspective. Each item is scored on a seven-point scale ranging from 1 (*strongly disagree*) to 7 (*strongly agree*). A higher score indicates greater public self-focus. The reliability and validity of this scale have been demonstrated [47].

#### Multidimensional Perfectionism Scale (MPS) [48]

The MPS was used to evaluate multidimensional facets of perfectionism. The MPS is a 35-item questionnaire that measures six dimensions of perfectionism: Concern over mistakes, Personal standards, Parental expectations, Parental criticism, Doubt about actions, and Organization. Each item is scored on a five-point scale ranging from 1 (*strongly disagree*) to 5 (*strongly agree*). A higher score indicates greater perfectionism. The MPS has demonstrated adequate reliability and high concurrent validity [48], and the Japanese version of the MPS has also been validated [49].

### Statistical analyses

Correlations were computed between “Fear of Obesity” and “Self-Esteem Based on Shape and Weight” subscale scores of the EDE-Q and the total scale scores of the RSES, PSS, MPI, and BDI-II. Partial correlations were computed to assess the relation between each factor subscale of the EDE-Q, RSES, PSS, MPS and BDI-II after controlling for “Fear of Obesity” or “Self-Esteem Based on Shape and Weight.”

## Results

The correlations between the two restructured EDE-Q subscales, “Fear of Obesity” and “Self-Esteem Based on Shape and Weight,” and the other psychological measurements on the RSES, PSS, MPI, and BDI-II. are shown in Table 5. Both subscales correlated significantly with RSES, PSS, MPS, and BDI-II scores. However, the partial correlations between “Fear of Obesity” and RSES, PSS, MPS, and BDI-II scores became nonsignificant after controlling for “Self-Esteem Based on Shape and Weight” scores. On the other hand, the relationships between “Self-Esteem Based on Shape and Weight” and RSES, PSS, MPS and BDI-II scores remained significant even after controlling for “Fear of Obesity”.

**Table 5:** Pearson’s correlation coefficients and partial correlations between the two factor subscale scores of the restructured EDE-Q and the total scale scores of RSES, PSS, MPS and BDI-II

	RSES	PSS	MPS	BDI-II
Fear of Obesity (controlling for Self-Esteem Based on Shape and Weight)	−0.36^***^/-0.09	0.30^***^/0.05	0.13^***^/-0.11	0.39^***^/0.09
Self-Esteem Based on Shape and Weight (controlling for Fear of Obesity)	−0.45^***^/-0.30^***^	0.40^***^/0.27^***^	0.30^***^/0.29^***^	0.50^***^/034^***^

The left of the slash displays pearson’s correlation coefficients

The right of the slash displays partial correlations after controlling for the other subscale of EDE-Q

*RSES* Rosenberg Self- Esteem Scale, *PSS* Public Self-Consciousness Scale, *MPS* Multidimensional Perfectionism Scale, *BDI-II* Beck Depression Inventory Second Edition

***p < 0.001

## Discussion

We explored the psychometric properties of the EDE-Q in Japanese undergraduate students. The factor structure of the EDE-Q derived from the present findings confirmed its internal consistency and convergent validity. However, the derived, restructured factors were somewhat different from the original four factors. “Restriction” and “Eating Concern” were retained as in the original EDE-Q factors, but “Shape Concerns” and “Weight Concerns” were combined into two different factors, “Fear of Obesity” and “Self-Esteem Based on Shape and Weight.” However, the distinction between “Fear of Obesity” and “Self-Esteem Based on Shape and Weight” indicated that only “Self-Esteem Based on Shape and Weight” was significantly associated with the measures that assessed psychopathology related to eating disorders.

Our exploratory factor analysis of the EDE-Q for these undergraduate students yielded four significant factors, which did not replicate the original EDE-Q subscales. Two derived subscales were different from the original ones: “Fear of Obesity” and “Self-Esteem Based on Shape and Weight” were distinct from the original “Weight Concern” and “Shape Concern,” whereas the factors “Restraint” and “Eating Concern” were replicated. However, scores of both the original and derived subscales and the global scores of the EDE-Q were higher for the female students than for the males. These findings support previous reports that young females show stronger concerns than males about their body image and eating restriction [29, 50].

Significant correlations between the derived subscale scores of the EDE-Q and eating disorder-related psychological measures of the EAT-26 and EDI-II provide further evidence of the convergent validity reported in Turkish and Spanish studies [51, 52].

“Fear of Obesity” and “Self-Esteem Based on Shape and Weight” scores were both highly correlated with the scores of the RSES, PSS, MPS, and BDI-II. However, after the partial correlation analysis, the significant correlations with “Fear of Obesity” disappeared after controlling for “Self-Esteem Based on Shape and Weight”, whereas “Self-Esteem Based on Shape and Weight” remained significant even after controlling for “Fear of Obesity.” This result demonstrated that “Self-Esteem Based on Shape and Weight” is a factor conceptually distinct from “simple” body dissatisfaction such as that assessed via “Fear of Obesity”. These findings are confirmed by the studies of Hrabosky et al. [22], Peterson et al. [13], and Grilo et al. [34], which emphasized the importance of the distinction between body image dissatisfaction and overvaluation of shape and weight with regard to the factor structure of the EDE-Q.

Comparing cross-cultural issues in terms of fear of obesity, Lee et al. [53] in Hong Kong reported that the majority of patients with Anorexia Nervosa (AN) in Hong Kong reported no conscious fear of becoming fat, which they termed “non-fat-phobic AN” (NFP-AN). Similarly, drive for thinness scores as assessed by the EDI-II were significantly lower for Japanese AN cases than in those in Western countries and showed scores similar to those of healthy controls [54]. On the other hand, Kusano et al. [33] found that female Japanese without eating disorders reported higher levels of body dissatisfaction and drive for thinness than their European counterparts, whereas Pike and Mizushima [55] found that female Japanese reported lower levels than a North American-referenced comparison. Thus, discrepancies between Japan and Western countries remain regarding the meaning of fear of obesity in the pathology of eating disorders [56]. This may be because the systemic analysis of data collected with the EDI in cross cultural comparisons indicated that both normal and eating-disordered samples [57] of non-Western people scored higher than Western people on virtually all EDI subscales.

In the present study, the mean scores of the original subscales of “Weight Concern” and “Shape Concern” on the EDE-Q were 1.95 and 2.25, respectively, which is compatible with the scores of European adolescents [29]. The scores for “Weight Concern” and “Shape Concern” are theoretically derived from the average of the corresponding items. It is interesting to note, however, that the mean score of “Fear of Obesity” appeared rather higher (2.75) than “Self-Esteem Based on Shape and Weight” (1.65). As described earlier, this may raise a question about the meaning of “fear of obesity” in the pathology of eating disorders between Japan and Western countries. It is necessary to further examine these new subscale scores among Japanese patient groups and their European counterparts.

A recent study conducted in Japan revealed three discernible groups of anorexic patients: fat-phobic typical AN, NFP-AN, and AN with no evidence of distortions related to body shape and weight (AN-NED) [58]. However, the features of “Fear of Obesity” and “Self-Esteem Based on Shape and Weight” among these three groups remain unknown, and it is also unclear if individuals who are specifically NFP-AN or AN-NED have essential AN characteristics. Although cases similar to NFP-AN have been reported in Western countries [59–61], it is necessary to examine if the “fat phobia” of these AN patients is due to pure “Fear of Obesity,” rather than to social self-consciousness assessed by “Self-Esteem Based on Shape and Weight.” Assessment using the restructured EDE-Q subscales may help reveal pathologies related to eating disorders in different cultures.

Overall, “Self-Esteem Based on Shape and Weight” may be a core feature of eating pathology related to the body and weight concerns of Japanese undergraduate students. Moreover, the findings provide empirical evidence for the clear distinction between “Fear of Obesity” and “Self-Esteem Based on Shape and Weight”.

### Limitations

Our sample comprised only undergraduate students, and the EDI-II was examined only with female students, which may limit the generalizability of the present findings.

In addition, comparison between Japanese and other Asian or ethnic samples was not done. It will be necessary in future studies to compare EDE-Q with other questionnaires regarding the evaluation of cultural differences. The factor structure of the EDE-Q should be examined across a wider range of ages and clinical samples to further assess validity. A confirmatory factor analysis (CFA) should also be conducted to confirm goodness of fit of the newly derived factor structure. Future research should consider those points and replicate these findings. However, in our view, the present results help clarify the psychological etiologies of eating disorders.

## Conclusions

In conclusion, we investigated the psychometric properties of the EDE-Q in Japanese undergraduate students. The distinction between “Fear of Obesity” and “Self-Esteem Based on Shape and Weight” may help facilitate future developments in the research and treatment of eating disorder patients from a cross-cultural perspective.

## Abbreviations

AN: Anorexia Nervosa
AN-NED: AN with no evidence of distortions related to body shape and weight
BDI-II: Beck Depression Inventory Second Edition
EAT-26: Eating Attitude Test-26
EDE-Q: Eating Disorder Examination-Questionnaire
EDI-II: Eating Disorders Inventory Second Edition
MPS: Multidimensional Perfectionism Scale
NFP-AN: Non-fat-phobic AN
PSS: Public Self-Consciousness Scale
RSES: Rosenberg Self-Esteem Scale
